# Prediction processes during multiple object tracking (MOT): involvement of dorsal and ventral premotor cortices

**DOI:** 10.1002/brb3.180

**Published:** 2013-10-03

**Authors:** Silke Atmaca, Waltraud Stadler, Anne Keitel, Derek V M Ott, Jöran Lepsien, Wolfgang Prinz

**Affiliations:** 1 Max Planck Institute for Human Cognitive and Brain SciencesLeipzig, Germany; 2 Technische Universität MünchenMunich, Germany; 3 Evangelisches Krankenhaus Königin Elisabeth Herzberge gGmbH, Institut für Diagnostik der EpilepsienBerlin, Germany

**Keywords:** Action prediction, dorsal premotor cortex, fMRI, multiple object tracking, perceptual event prediction, predictive forward models, ventral premotor cortex

## Abstract

**Background:**

The multiple object tracking (MOT) paradigm is a cognitive task that requires parallel tracking of several identical, moving objects following nongoal-directed, arbitrary motion trajectories.

**Aims:**

The current study aimed to investigate the employment of prediction processes during MOT. As an indicator for the involvement of prediction processes, we targeted the human premotor cortex (PM). The PM has been repeatedly implicated to serve the internal modeling of future actions and action effects, as well as purely perceptual events, by means of predictive feedforward functions.

**Materials and methods:**

Using functional magnetic resonance imaging (fMRI), BOLD activations recorded during MOT were contrasted with those recorded during the execution of a cognitive control task that used an identical stimulus display and demanded similar attentional load. A particular effort was made to identify and exclude previously found activation in the PM-adjacent frontal eye fields (FEF).

**Results:**

We replicated prior results, revealing occipitotemporal, parietal, and frontal areas to be engaged in MOT.

**Discussion:**

The activation in frontal areas is interpreted to originate from dorsal and ventral premotor cortices. The results are discussed in light of our assumption that MOT engages prediction processes.

**Conclusion:**

We propose that our results provide first clues that MOT does not only involve visuospatial perception and attention processes, but prediction processes as well.

## Introduction

During visual perception, sensory input is constantly disrupted due to eye blinks, saccadic eye movements, and outside world occluders. As a consequence, there is a perpetual loss of visual information, particularly critical during the observation of moving entities. Yet, the human brain manages well to compensate this information loss, for example, sustaining object identities through (brief) occlusions during the attentive tracking of moving objects (Scholl and Pylyshyn 1999; Franconeri et al. 2006). It has been suggested that identity correspondence is maintained based on information regarding object surface features and spatiotemporal continuity (e.g., Hollingworth and Franconeri 2009; also see below).

In the multiple object tracking (MOT) paradigm, participants have to keep track of several moving targets among a similar number of moving distractor objects. These objects (targets and distractors) do not bear any distinguishing characteristics except for different (premotion) starting locations. Thus, target identities are maintained through the continuous processing of spatiotemporal information, constantly updating target locations. In this study, we raise the question of whether past and current spatiotemporal target characteristics are used to extrapolate future target locations via sensorimotor prediction processes.

The human premotor cortex (PM) has been implicated to be a key neural substrate for the prediction of motor acts (e.g., Stadler et al. 2011) and dynamic perceptual events (Wolfensteller et al. 2007). Accordingly, we expected the PM to be engaged during MOT. In the following sections, we will thoroughly introduce the MOT paradigm, illustrate the role of the PM in sensorimotor prediction, and reflect on previous experimental evidence speaking in favor of the employment of prediction processes during MOT. We will conclude the Introduction with our hypothesis and experimental rationale.

### MOT paradigm

**Figure 1:**
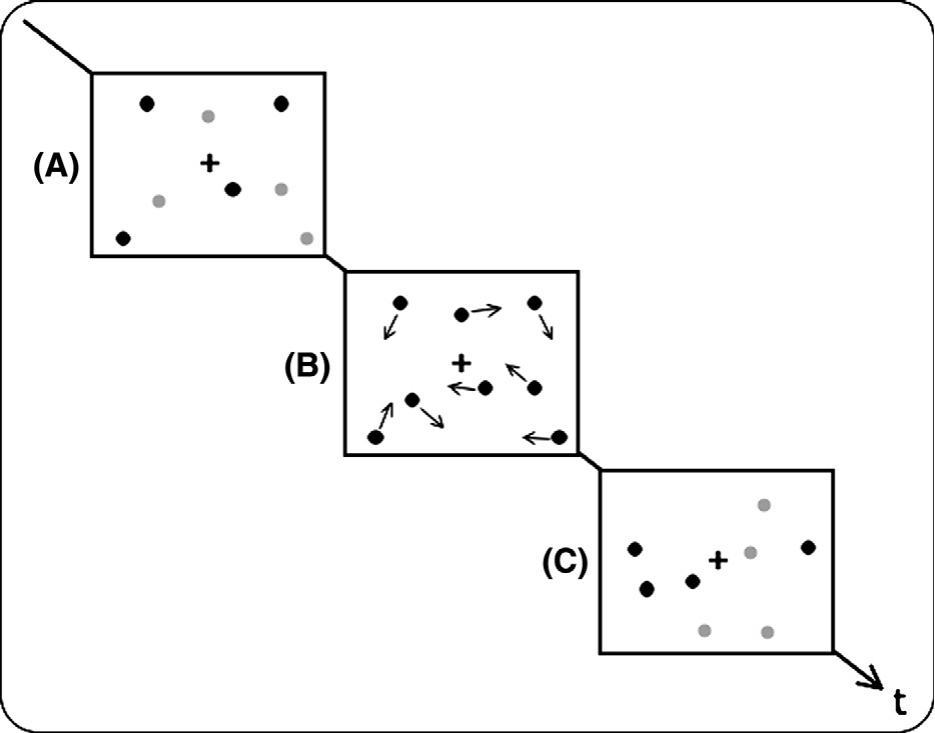
Depicted is the course of a trial in a typical MOT task (e.g., Pylyshyn and Storm 1988). Participants see a small number of objects (e.g., eight circles). (A) In the *target presentation period*, a subset of these objects (e.g., four) is marked as targets. (B) In the *motion period*, markings are erased, rendering objects identical in appearance. All objects move about the screen for a short period of time, for example, 10 sec, usually constrained to a predetermined *motion area*. (C) In the subsequent *target identification period*, participants are asked to identify the targets.

The MOT paradigm is a cognitive task originally developed to study visual attention (Pylyshyn and Storm 1988), targeting the question of whether several identical, moving objects can be tracked in parallel despite the finding of one locus of visual attention (Posner et al. 1980). A typical MOT task has the following characteristic (see Fig. 1): participants see a small sample of objects (e.g., eight circles). In the *target presentation period*, a subset of these objects (e.g., four) is marked as targets. Subsequently, all objects are indistinguishable and move around the screen during the *motion period* that lasts, for instance, 10 s. Object motion is usually constrained to a predetermined subarea of the screen, the *motion area*. After the motion has stopped, participants are asked to identify the targets (*target identification period*).

As demonstrated repeatedly, humans can reliably track up to four or five objects (Pylyshyn and Storm 1988; Scholl and Pylyshyn 1999; Scholl et al. 2001). Tracking performance is modulated by a number of factors determining cognitive demands, such as object velocity (Alvarez and Franconeri 2007), number of nontargets (Sears and Pylyshyn 2000), length of motion period (Pylyshyn 2004), and visual angle of the overall display (Intriligator and Cavanagh 2001).

Motion trajectories are typically rendered based on an algorithm resulting in “Brownian motion like” object motion (cf. Pylyshyn and Storm 1988; “Brownian motion” is a term used in physical chemistry to describe the movement of particles in suspension, resulting from collisions with rapidly moving atoms or molecules). There are restrictions regarding sudden and large velocity changes (a factor referred to as *object inertia*, Pylyshyn 2004), giving object motion a certain appearance of “biologicity.” Moreover, motion trajectories are affected by object “behavior”: in the incidence of intersection, two objects can overlap with each other (Pylyshyn 2004), bounce off each other (Bahrami 2003), or go round each other (Alvarez and Franconeri 2007). Such constraints restrict the set of possible spatial coordinates to which an object can proceed from one frame to another. However, from the remaining set, coordinates are usually chosen in an arbitrary manner, yielding essentially unpredictable object motion.

### Cognitive processes during MOT

Behavioral results on MOT cannot readily be explained by “spotlight” theories of attention (Posner 1980). Rather, Yantis (1992) found empirical evidence that target objects are “grouped,” that is, cognitively represented as if belonging to one virtual object. During tracking, instead of continuously shifting the locus of attention back and forth between objects, target identity is supposedly maintained via a holistic representation in the form of said virtual object.

Furthermore, Pylyshyn and Storm (1988) suggested that object identity is maintained through “mental reference tokens.” In an early stage of visual perception, salient objects in a visual display can be “indexed,” a mechanism that individuates and keeps track of said objects without the necessity to categorize or conceptualize them (Pylyshyn 2001). On a similar notion, Kahneman and colleagues suggested the existence of *object files*, that is, temporary visual representations of real world objects (Kahneman and Treisman 1984; Kahneman et al. 1992). Object files store information on object surface features, such as shape or texture, and spatiotemporal characteristics (Mitroff and Alvarez 2007). Depending on their availability and reliability in a given situation, both types of information can be used to maintain object correspondence in the incidence of brief occlusions of an observed moving object, (Hollingworth and Franconeri 2009; Papenmeier et al. in press). However, in situations where no distinguishing surface information is available (such as in the MOT paradigm), spatiotemporal information appears to be of key significance to the maintenance of object file representations. Indeed, while random changes in object shape or object color did not impair tracking performance (Bahrami 2003; unless, for instance, targets and distractors swapped colors during occlusion, see Huff et al. 2011), the manipulation of spatiotemporal information did lead to tracking impairment (Franconeri et al. 2006; also see below). Furthermore, object trajectories have been demonstrated to be a crucial parameter in target-distractor discrimination. When the MOT movement algorithm was altered in a way that resulted in an interdependence of target and distractor trajectories (e.g., “behaving” as if chasing each other), tracking performance declined significantly (Suganuma and Yokosawa 2006).

Importantly, we propose that object identity is not only sustained based on past motion trajectories, but that spatiotemporal information is also used as a feedforward function. Should our assumption hold true, then prediction processes should be indicated by PM activation during MOT, as will be elaborated in the following section.

### Prediction processes and the PM

The premotor cortex, as its name implicates, is crucially involved in the planning and preparation of motor acts (for a meta-analysis, see Grèzes and Decety 2001). Interestingly, some parts of the PM (particularly those located in the inferior frontal gyrus [IFG]), not only show involvement in processes of action control, but during the observation of motor acts as well (Rizzolatti and Craighero 2004). During action observation, these areas have been suggested to translate visual codes into action codes, providing a neurophysiological link between visual perception and action control (Rizzolatti et al. 2001; Rizzolatti and Sinigaglia 2010). More precisely, it appears that prediction processes, as employed during action control (e.g., generating short-term templates of expected sensory consequences of an action, see Schubotz 2007), are also exploited during action perception (Blakemore and Decety 2001).

Importantly, there is accumulating evidence that PM activation reflects the simulation and prediction of yet to be performed actions (Schubotz and von Cramon 2004; Stadler et al. 2011, 2012). Such “emulations” of others’ actions (Schubotz 2007) are not necessarily limited to an observer's ability to reproduce the observed or predicted action with their own motor system, nor do the observed actions have to be of human origin in the first place (Cross et al. 2011a,b). Rather, Schubotz (2007) proposed that said emulations are used “*by default* in a simulation mode for predictions of observable events *of any kind* as long as they take place within several seconds” (Schubotz 2007, p. 211; italics added for emphasis). That is, even in the absence of motor requirements, the PM functions as an “internal forward model of environmental dynamics” (Schubotz and von Cramon 2003, p. S124), modeling dynamic sensory patterns based on sequential event characteristics (Schubotz and von Cramon 2003, 2004; Schubotz 2007; Wolfensteller et al. 2007).

The following section will review previous experimental evidence that, we argue, speaks in favor of the employment of prediction processes and PM involvement during MOT.

### MOT, sensorimotor prediction, and the PM

In a behavioral MOT study, Franconeri et al. (2006) manipulated the location of object reappearance after object motion had been briefly occluded. Tracking performance was impaired when objects exited the occluder at unexpected locations (e.g., shifted by several object diameters on the vertical axis). Similarly, Graf et al. (2007) modulated the continuity perception of human movement with another occluder paradigm. Watching short sequences of familiar actions, participants’ task was to detect changes in specific movement parameters after occlusion. Behavioral performance varied as a function of the degree to which occluder length matched the time gap in the occluded movement (both systematically manipulated), with highest performance for perfect matches. That is, in both studies (Franconeri et al. 2006; Graf et al. 2007), the manipulation of spatiotemporal parameters of an observed motion hampered motion perception. The results by Graf et al. (2007) have been taken to demonstrate real-time simulation of observed actions. As a consequence, experimental alterations of the observed actions led to violations of anticipated visuospatial input. We propose that the findings by Franconeri et al. (2006) were based on similar cognitive processes. Furthermore, Stadler et al. (2011) conducted a functional magnetic resonance imaging (fMRI) experiment, adopting the Graf occlusion paradigm. The authors compared brain activation elicited by a simulation task to brain activation evoked by cognitive control tasks, for example, a memory task. Results suggest significantly more (left hemispheric) dorsal premotor cortex (PMd) activation during the employment of prediction processes in the occluder phase, compared to other cognitive mechanisms (e.g., memory processes).

In another behavioral study, Trick et al. (2006) found interferences between MOT and action execution. Subjects performed (1) a standard MOT task, (2) a standard MOT task while additionally performing three-finger tapping sequences, (3) a standard MOT task while additionally articulating three-syllable sequences. MOT performance was significantly more impaired during additional finger tapping, suggesting that finger tapping and object tracking share cognitive resources and respective neural substrates, possibly the PM. In a meta-analysis, Schubotz and von Cramon (2003) studied activation patterns in the PM during performance of cognitive tasks demanding object-related attention (e.g., observation and denotation of familiar tools, Grafton et al. 1997), rhythm-related attention (e.g., detection of rhythm violations, Schubotz and von Cramon 2001), and spatial attention (e.g., trajectory predictions of single moving dots, Chaminade et al. 2001). The authors found that spatial attention rather elicited activation in dorsal parts of the PM (PMd), while rhythm and object-related attention rather elicited activation in ventral parts of the PM (PMv). The same meta-analysis discussed somatotopic activation patterns of the PM, revealing PMd involvement in eye movement control, and PMv involvement in execution, observation and imagery of hand and finger movements (Buccino et al. 2001; Schubotz and von Cramon 2003; Schubotz 2007). Such task- and body part-specific activations could explain why MOT was affected by finger tapping: because the brain regions (presumably subregions of the PM) that are engaged in the planning of rhythmic, spatially defined actions (assuming that tapping sequences are spatially coded), as well as the execution of these actions by means of finger and concomitant eye movements, are also engaged in MOT.

Previous fMRI studies have investigated brain activation during MOT (Culham et al. 1998, 2001; Jovicich et al. 2001; Howe et al. 2009). All four studies compared an MOT condition (subjects had to track a subset of 2–5 out of 8–10 objects) with a passive viewing condition (moving circles without tracking instruction), revealing several loci of activation in the parietal cortex, such as the anterior and the posterior intraparietal sulcus and the superior parietal lobule. Importantly, the contrast [MOT > passive viewing] also showed activation in frontal regions, namely in the dorsolateral frontal cortex (DLFC; Culham et al. 1998, 2001; Howe et al. 2009). Furthermore, there was activation associated with tracking load (increasing activation with increasing number of tracked objects) in the left inferior precentral sulcus (Culham et al. 1998, 2001; Jovicich et al. 2001).

Activations in the DLFC have been interpreted to refer to the frontal eye fields (FEF). FEF are crucially involved in oculomotor control (Paus 1996) and processes of spatial attention (Corbetta 1998; also see Discussion for a review of FEF involvement). Activation in the FEF was thus attributed to generation and suppression of involuntary eye movements and attention shifts during MOT (Culham et al. 1998, 2001; Howe et al. 2009). Furthermore, Jovicich et al. (2001) interpreted activation in the DLFC to represent an area they named “primary motor area,” assumed to reflect motor preparations prior to executing a response in form of a button press. Indeed, MOT required a response in the end of each trial, passive viewing did not (Jovicich et al. 2001). The authors discussed that this activation in the primary motor area might have concealed activation in the adjacent FEF. In turn, we propose that activation in the DLFC, as has been found by all four studies, refers to the FEF-adjacent PMd, partly concealed by FEF activation. Similarly, we propose that previously found activation in the inferior precentral sulcus (Culham et al. 2001; Jovicich et al. 2001) indicates involvement of the PMv, possibly reflecting sensorimotor prediction processes.

That is, in accordance with previous behavioral results (Franconeri et al. 2006; Trick et al. 2006) and found brain activation maxima (Culham et al. 1998, 2001; Jovicich et al. 2001; Howe et al. 2009), *we expected activation in the DLFC during MOT*. Such brain activation would be in accordance with (yet no definite proof of) the recruitment of prediction processes during MOT.

### Hypothesis and experimental approach

In the current study, we aimed to provide first evidence for the employment of sensorimotor prediction processes during the parallel tracking of several identical objects following arbitrary motion trajectories (MOT paradigm). We operated under the rationale that *prediction processes should be reflected by premotor activation during MOT*. While potential findings of activation in the DLFC would neither allow for the inevitable conclusion of PM involvement, nor for this PM involvement to be an indicator of prediction processes, we took experimental measures to smooth the way for a respective result interpretation.

In order to test our hypothesis, we adopted a standard MOT task (Pylyshyn and Storm 1988) where participants had to track either two or three out of eight identical objects (for a detailed description, see Methods section). As control condition, we implemented a cognitive task that allowed application of identically the same stimulus material in both conditions, with an initial cue signaling which task to execute. With this experimental design, we ensured identical visual input and minimized differences between MOT and control condition in regard to level of vigilance and attentional load. We also circumvented the problem of response preparation as a source of premotor activation (Jovicich et al. 2001), as a response was required in both conditions.

As described above, previous fMRI studies on MOT (Culham et al. 1998, 2001; Jovicich et al. 2001; Howe et al. 2009) found increased activation in the DLFC. This activation was interpreted to originate from the FEF, a region anatomically adjacent to PMd (Paus 1996; Schubotz and von Cramon 2001). Since a major concern was the dissociation between the FEF and the PM, we sought to considerably reduce later confusions regarding the origin of potential activations. To that end, we (1) conducted a behavioral prescreening and selected participants with minimal eye movements, and (2) functionally localized the FEF and later masked the main contrast (MC) with localizer activation.

## Methods

### Participants

Participants were recruited via the subject pool of the Max Planck Institute for Human Cognitive and Brain Sciences (MPI-CBS) in Leipzig, Germany. Out of 23 that took part in a prescreening (procedure described below), the 13 participants with the least eye movements and concomitant highest behavioral performance were invited to participate in the fMRI scanning. The data of two participants were later removed from further analyses due to error rates of >25% during fMRI scanning. The remaining 11 participants ranged in age from 22 to 33 (mean age 26.9 years, three female).

### Experimental conditions: MOT and luminance changes

#### Stimuli

Stimuli featured eight identical objects (white squares, roughly 0.2° of visual angle) and a centrally positioned fixation cross (roughly 0.2° of visual angle). In the *motion period* (see below, Course of trials), these objects moved around for 6000 msec in an arbitrary fashion, confined by the *motion area*, a gray square in the center of the computer screen (roughly 7° of visual angle). Motion trajectories were calculated online. The motion algorithm was based on the one used by Sears and Pylyshyn (2000). Objects moved at a predetermined, constant velocity. In order to avoid ambiguities in respect to object identities, some restrictions were put into place regarding “object behavior” (Pylyshyn and Storm 1988). Should an object collide with the border of the motion area, the fixation cross, or another object, it “bounced off,” reversing the perpendicular component of its velocity. This procedure led to abstract and arbitrary object motion, resembling “Brownian motion” (Sears and Pylyshyn 2000; also see above, MOT paradigm”). In addition, objects simultaneously underwent 2–6 luminance changes (LUM) during the motion period. Each luminance change lasted for 500 msec, with a minimum of 300 msec between two changes. Note that these stimulus characteristics (object motion, LUM) were precisely the same for both conditions.

#### MOT condition

Participants had to track a subset of either two or three out of the eight identical objects throughout the motion period (representing difficulty level 1 and 2, respectively).

#### Luminance changes (LUM condition)

As control condition, participants were asked to count the number of LUM. Luminance values (8-bit grayscale) changed either from 255 to 210 (difficulty level 1), or from 255 to 220 (difficulty level 2), with the latter being less salient und thus representing a higher degree of difficulty.

#### Course of trials

(1) In the initial *target presentation period*, objects appeared in a random position within the motion area, with the restriction that they must not be directly adjacent to or overlap with the border of the motion area, the fixation cross, or another object. The target presentation period functioned as a *task cue*. Either a subset or all objects were “marked,” that is, they changed color from white to red. Marking two or three of the eight objects indicated that in the following motion period, the marked objects had to be tracked (MOT condition). When all eight objects were marked, participants had to count LUM (LUM condition). Markings lasted throughout the duration of the target presentation period, which was jittered (1750, 2000, 2250, 2500, 3000 msec). Subsequently, there was a short still period of 1000 msec where participants saw the same display of eight objects without the markings. (2) In the following *motion period*, objects were indistinguishable and moved around within the motion area for 6000 msec while simultaneously undergoing several changes in luminance. (3) After the motion had stopped, a solution was presented for 2000 msec (*target identification period*). In the MOT condition, a subset of objects was marked, corresponding to the number of targets in the respective trial. Participants had to indicate via button presses whether the marked objects were targets or not. In 50% of cases, the offered solution was incorrect, differing by one object from correct target identities. In the LUM condition, the fixation cross was replaced by an Arabic digit. Participants had to indicate via button presses whether the presented number equaled the number of LUM or not. In 50% of cases, the offered solution was incorrect, differing by (+/−) one from correct number of LUM. There were intertrial intervals (ITIs) of 4000 msec.

### FEF localizer task

Previous studies have associated the FEF with oculomotor control and shifts in spatial attention during visual processing (Anderson et al. 1994; Paus 1996; Corbetta 1998; Pierrot-Deseilligny et al. 2004). Accordingly, in order to localize participants’ FEF, we implemented an FEF localizer (FEF-L; cf. Garg et al. 2007). The display featured the same motion area (roughly 7° of visual angle) and fixation cross (roughly 0.2° of visual angle) as MOT and LUM. Fixation periods (FIX) alternated with saccade periods (SACC), lasting 15 sec, respectively. During FIX, the fixation cross was presented centrally. During SACC, the fixation cross randomly appeared in one of the four corners of the motion area, changing location in 1500 msec intervals. Participants’ task was to rapidly move their eyes toward the location of appearance. Such exogenous, visually guided saccades comply with eye movements that might occur during MOT despite the instruction to fixate the centrally presented cross. That is, with the specific design of the FEF-L task, we aimed to elicit FEF activation associated with eye movements that bear characteristics similar to those possibly occurring during MOT (also see Discussion below).

## Experimental Procedure

Both prescreening and fMRI-recording took place at MPI-CBS. All participants had normal or corrected-to-normal vision, gave written consent, and received monetary reward for their participation.

### Prescreening

Aiming to confine eye movements during the experiment in order to reduce FEF involvement to a minimum, we conducted a behavioral prescreening. During MOT, participants’ eye movements were recorded using a remote corneal reflection eye tracker (Tobii 1750, Stockholm, Sweden; software ClearView 2.7.1; sampling rate: 50 Hz). Participant selection was then based on both behavioral performance and the occurrence of saccades.

### fMRI scanning

During scanning, participants attended to 100 trials of stimuli (50 MOT, 50 LUM), presented at 25 frames per second (60 Hz refresh rate) with a resolution of 1024 × 768 pixels. The software “Presentation” (Neurobehavioral Systems™, Albany, CA) was used for stimulus presentation and response recording. Using a back projection system, stimuli were displayed above participants’ eyes via a mirror reflecting an LCD projection onto a screen placed behind the magnet. Including all measured sequences, scanning time did not exceed 50 min.

## fMRI data acquisition and analysis

### fMRI data acquisition

Functional images were acquired on a 3T BRUKER MedSpec 30/100 system (Bruker Corporation, Billerica, MA), equipped with a standard birdcage head coil. Functional images were collected with a single shot gradient echo-planar imaging (EPI) sequence with the following parameters: echo time TE = 25 msec, flip angle 90°, repetition time TR = 2000 msec, acquisition bandwidth 100 kHz. Twenty-six axial slices were taken in an interleaved fashion (pixel matrix = 64 × 64 and in-plane resolution = 3 × 3 mm, resulting in a field of view of 19.2 cm, a slice thickness of 4 mm, and an interslice gap of 1mm), oriented parallel to the bicommissural plane (AC-PC). The total number of functional scans collected per participant was 780 for the experimental conditions and 233 for the FEF-L. Additionally, three-dimensional (3D) high-resolution whole brain images were acquired from each subject (MP-RAGE sequence, 160 slices, 1 mm thickness) in a separate session on a 3T Siemens MAGNETOM TIM Trio (Siemens AG, Munich and Berlin, Germany), used to align the functional data slices onto a 3D stereotactic coordinate reference system.

### fMRI data preprocessing

All fMRI data analyses were carried out using the SPM8 software package (Wellcome Department of Imaging Neuroscience, London, U.K.) with Matlab 7 (Mathworks, Natick, MA). After EPI volumes were corrected for motion, distortion, and slice timing, they were realigned, unwarped, normalized to the Montreal Neurological Institute (MNI) template (3 × 3 × 3 mm resolution), and spatially smoothed (8 mm).

### fMRI data first-level analysis

Each motion period (time between end of still period and beginning of target identification period, see above) was modeled as a boxcar spanning the length of 6000 msec, convolved with the standard hemodynamic response function, representing activation during MOT and LUM, respectively. Accordingly, a design matrix was fitted with regressors for MOT and LUM. Trials that showed erroneous behavioral performance were modeled just as regular MOT and LUM trials, yet labeled as JUNK. JUNK and BASELINE (modeled as a boxcar spanning the duration of 4000 msec ITIs) entered the analysis as additional regressors. For first-level analysis, contrast images were computed combining the parameter estimates of the corresponding experimental conditions (MOT, LUM).

For the FEF-L, a design matrix was fitted with regressors for FIX and SACC, each modeled as a boxcar with a duration of 15 s and convolved with the standard hemodynamic response function. Computing contrast images combining the parameter estimates of FIX and SACC, effects of the two regressors were compared to each other resulting in FEF-L activation. This was done on the group level due to the circumstance that individual subjects showed large variations in activation strength. While a few outliers did not show any frontal activation related to the FEF-L with the current analysis parameters, in other participants, changing the significance level to a value higher than *P*_uncorrected_ < 0.001 resulted in such massive brain activation that it no longer could be called meaningful. However, we did not feel comfortable with applying different analysis parameters to different participants. As a consequence, we performed the analyses on the group level, reasoning that, by following this more conservative way, we would end up excluding rather too much activation as being FEF related than not enough.

### fMRI data second-level analysis

For group analysis, said contrast images were fed into one-sample *t*-tests, testing found between-condition differences against zero (Holmes and Friston 1998). The main contrast (MC) examined differences in activation maxima between the conditions MOT and LUM, [MOT > LUM]. The FEF-L mask was acquired by computing the contrast between SACC and FIX, [SACC > FIX]. FEF-L was used as an exclusive mask to eliminate activation related to oculomotor control and stimulus-driven attention shifts from the MC. Both contrasts were evaluated in whole brain analyses. The MC was evaluated at the *P*_uncorrected_ < 0.001, *k* = 10 voxel threshold. Only results that reached a significance level of *P*_FDR-corrected_ < 0.001 (i.e., corrected for false-discovery rate) will be discussed below. Note that exceptions were made for two clusters that were deemed particularly worthy to be discussed in light of the current study, despite the fact that they did not reach *P*_FDR-corrected_ < 0.001. The FEF-L mask was evaluated at the *P*_uncorrected_ < 0.001, *k* = 0 voxel threshold. We intentionally set the voxel threshold as low as possible in order to ensure that no FEF activation would be dismissed. The resulting activations were saved as an image file, and used to be applied as an exclusive mask to the MC. Coordinates of found brain activations and corresponding anatomical structures are summarized in Tables 1 and 2. Brain activations were anatomically localized with aid of SPM8's Anatomy Toolbox (Eickhoff et al. 2005), double checked, and corrected (where applicable) by expert neuroanatomist D. V. M. Ott, M.D. (coauthor to this paper).

**Table 1:** Effects of simultaneous tracking of two and three objects (average)

H	AR	*x*	*y*	*z*	cs	*t*
R	*Middle temporal gyrus	54	−55	13	3770	12.84
R	*Supramarginal gyrus	45	−31	43		11.85
R	*Middle occipital gyrus	42	−73	25		10.10
L	*Precentral gyrus (BA6)	−15	−10	67	414	8.82
L	*Superior frontal gyrus (BA6)	−21	5	49		8.73
L	*Precentral sulcus (BA6)	−21	−7	55		8.55
R	*Precentral gyrus (BA6)	21	−10	61	305	9.01
R	*Precentral sulcus (BA6)	18	−7	52		7.94
R	*Precentral gyrus (BA6)	33	−10	58		7.92
L	Cerebellum	−15	−52	−50	240	7.65
L	Cerebellum	−42	−43	−29		7.45
L	Fusiform gyrus	−27	−43	−14		7.06
R	Precentral gyrus, pars opercularis of IFG (BA44)	51	5	31	55	6.19
R	Precentral gyrus, pars opercularis of IFG (BA44)	54	2	22		5.26
R	N/A	33	2	−17	55	8.37
R	Olfactory cortex	18	11	−20		5.28
L	Superior temporal gyrus	−57	−19	4	36	7.54
R	Fusiform gyrus	30	−31	−23	33	5.77
R	Parahippocampal gyrus	27	−25	−23		5.66
L	Anterior cingulate cortex	−3	11	25	32	6.11
R	N/A	12	23	19		5.82
R	N/A	6	14	22		5.04
R	Superior temporal gyrus	42	−28	10	27	7.41
L	Cerebellum	−18	−64	−17	26	5.49
L	Cerebellum	−21	−67	−20		5.37
R	Mid orbital gyrus	9	50	−11	25	5.00
R	N/A	18	47	−8		4.84
L	Olfactory cortex	−6	11	−17	23	5.39
R	N/A	3	2	−14		4.90
R	Temporal pole	48	17	−17	23	6.22
R	N/A	24	17	10	20	5.09
R	N/A	21	−49	22	19	5.96
L	N/A	−15	−28	34	18	7.44
L	Precentral gyrus, pars opercularis of IFG (BA44)	−51	5	25	18	5.76
R	N/A	36	−1	1	16	4.93
R	Fusiform gyrus	33	−43	−11	12	4.75
R	Cerebellum	12	−73	−50	12	5.79
L	N/A	−57	8	−17	11	5.03
L	Middle temporal gyrus	−60	−1	−17		4.51

In a whole brain analysis, evaluated at *P*_uncorrected_ < 0.001, *k* = 10 voxel threshold, we compared brain activation during multiple object tracking (MOT) with brain activation elicited by the control condition (detection of LUM). For the main contrast, [MOT > LUM] was masked with activation of the FEF localizer (FEF-L, for activation maxima, see Table 2). Table 1 lists the found brain activation maxima for [MOT > LUM] (exl. FEF-L). H, Hemisphere; AR, anatomical region according to SPM's Anatomy Toolbox Probability Maps, if applicable corrected by expert neuroanatomist and coauthor D. V. M. Ott, M.D.; BA, brodmann area; *x*/*y*/*z*, MNI coordinates; cs, cluster size; *t*, *T*-value; IFG, inferior frontal gyrus.

* Results that reached a significance level of *P*_FDR-corrected_ < 0.001.

**Table 2:** Effects of visually guided oculomotor control (FEF localizer task)

H	AR	*x*	*y*	*z*	cs	*t*
R	*SPL, precuneus	9	−61	58	358	9.73
R	*Superior parietal lobule	18	−67	52		7.85
R	*Superior parietal lobule	24	−58	55		7.34
R	*Inferior parietal lobule	33	−43	49		6.66
L	Inferior Parietal Lobule	−27	−52	52	251	8.02
L	Superior parietal lobule	−18	−61	55		7.84
L	Precuneus	−12	−70	49		5.64
L	Calcarine gyrus	−12	−82	4	238	8.33
R	Calcarine gyrus	9	−82	4		6.91
R	Superior frontal gyrus	24	−4	52	137	7.63
R	Precentral gyrus	42	−4	49		6.86
R	Middle occipital gyrus	33	−76	31	113	7.20
R	Middle occipital gyrus	33	−73	19		6.38
L	Precentral gyrus	−27	−4	61	77	6.07
L	Precentral gyrus	−30	−7	58		5.99
L	Cerebellum	−33	−46	−50	23	5.89
L	Cerebellum	−15	−55	−47	9	6.28
R	Superior temporal gyrus	57	−40	19	7	4.74
R	Supramarginal gyrus	66	−31	25	4	4.92
R	Middle temporal gyrus	57	−43	7	4	4.48
L	Inferior parietal lobule	−45	−37	40	3	4.44
R	Middle occipital gyrus	42	−79	1	3	4.42
R	Inferior occipital gyrus	39	−82	−2		4.25
L	Cerebellum	−42	−55	−35	2	4.31

In a whole brain analysis, evaluated at *P*_uncorrected_ < 0.001, *k* = 0 voxel threshold, we compared brain activation during saccade execution compared to brain activation during fixation [SACC > FIX]. The resulting activation maxima of this FEF localizer (FEF-L), as listed in Table 2, were applied as an exclusive mask to [MOT > LUM]. H, hemisphere; AR, anatomical region according to SPM's Anatomy Toolbox Probability Maps; *x*/*y*/*z*, MNI coordinates; cs, cluster size; *t*, *T*-value; FEF, frontal eye fields.

* Results that reached a significance level of *P*_FDR-corrected_ < 0.001.

## Results

### Behavioral results

As behavioral performance, we compared number of correct responses out of 25 per condition: MOT2 (mean: 23.10; SD: 1.92), MOT3 (mean: 22.36; SD: 1.43), LUM1 (mean: 23.18; SD: 1.89), and LUM2 (mean: 22.09; SD: 2.91). A within-subjects 2 × 2 analysis of variance (ANOVA) with the factors Condition (MOT vs. LUM) and Task Difficulty (Level 1 vs. Level 2) was computed on the amount of correct responses. There was a significant main effect for the factor Task Difficulty, *F*(1,10) = 6.780, *P* < 0.05, indicating that our manipulation of task difficulty worked as intended. Neither the main effect for the factor Condition (*F*(1,10) = 0.018, *P* = 0.895) nor the two-way interaction of Condition × Task Difficulty (*F*(1,10) = 0.151, *P* = 0.706) reached significance, indicating that comparable cognitive demands were required by MOT and LUM and that task difficulty did not depend on condition.

### Imaging results

#### MC: main effect of condition [MOT > LUM]

**Figure 2:**
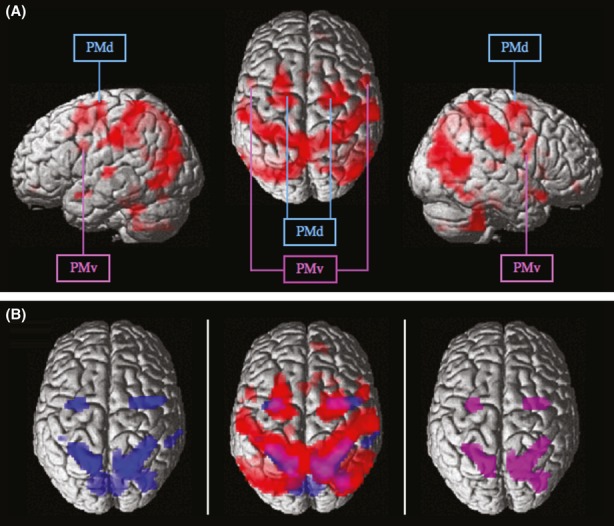
(A) Brains are seen from three different angles. Left: side view of the left hemisphere. Middle: dorsal view (neurological convention) of both hemispheres, with the anterior side of the brain pointing upwards. Right: side view of the right hemisphere. All three brains depict regions that were more activated during MOT compared to LUM, [MOT > LUM] (*P*_uncorrected_ < 0.001, *k* = 10 voxel), while FEF-L (*P*_uncorrected_ < 0.001, *k* = 0 voxel) was applied as an exclusive mask. [MOT > LUM] (excl. FEF-L) revealed frontal activations in BA6 (*P*_FDR__-corrected_ < 0.001), comprising the precentral gyrus (bilaterally), the precentral sulcus (bilaterally), as well as the left superior frontal gyrus (possibly merging into BA8). These activations in BA6 are assumed to refer to the dorsal premotor cortex (PMd, marked in blue). Further frontal activations were found bilaterally in BA44, in the pars opercularis of the inferior frontal gyrus (IFG), assumed to reflect involvement of the ventral premotor cortex (PMv, marked in pink). Furthermore, a large cluster spreading bilaterally through the temporal, parietal, and occipital cortices was revealed, with activation maxima in the superior and middle temporal gyri (bilaterally), the supramarginal gyrus (right hemisphere), and the middle occipital gyrus (right hemisphere). For coordinates of all activation maxima, see Table 1. (B) All three brains are seen from the dorsal view (neurological convention), with the anterior side of the brain pointing upwards. Left: Brain activations during performance of the FEF-L task. Depicted are those regions that were more activated during saccade execution compared to fixation, [SACC > FIX] (*P*_uncorrected_ < 0.001, *k* = 0 voxel), also referred to as the “FEF-L mask” (for coordinates of activation maxima, see Table 2). Middle: Depicted are two contrasts, [MOT > LUM] (*P*_uncorrected_ < 0.001, *k* = 10 voxel) in red and the FEF-L mask (*P*_uncorrected_ < 0.001, *k* = 0 voxel) in purple. Brain regions that showed activations in both contrasts are represented in lilac. Right: Depicted are only those brain regions that showed activations in both contrasts, [MOT > LUM] (*P*_uncorrected_ < 0.001, *k* = 10 voxel) and the FEF-L mask (*P*_uncorrected_ < 0.001, *k* = 0 voxel). Color brightness is not mapped to activation intensity, but corresponds to the locations of activations. The more transparent an activation, the more distant it is from the brain surface.

In order to reveal brain activation specific to the MOT task, we contrasted the MOT condition with detection of LUM (LUM condition). To disentangle activation related to eye movement control from task specific activation, FEF-L was applied as an exclusive mask. Following this procedure, the MC, [MOT > LUM] (excl. FEF-L), revealed bilateral frontal activations (Fig. 2), namely in the precentral gyrus, the precentral sulcus, the pars opercularis of IFG, and the left superior frontal gyrus. Furthermore, we found bilateral activation maxima in the middle temporal gyrus and the superior temporal gyrus, as well as in the right supramarginal gyrus, and the right middle occipital gyrus, and various activations throughout the brain that will not be further discussed. See Tables 1 and 2 for all activation maxima of the MC and the FEF-L mask, respectively.

## Discussion

Proposing that MOT employs sensorimotor prediction processes, this study investigated the recruitment of the DLFC (presumably the PM), taken as a neural correlate of predicting dynamic events during object tracking.

Previous brain imaging studies on MOT (Culham et al. 1998, 2001; Jovicich et al. 2001; Howe et al. 2009) focused on neural substrates of visuospatial attention, attentional load, spatial memory, and cognitive tagging of individual objects. These studies found a network of activations, dominantly in the parietal and the frontal cortices. Shedding light on these results from the perspective of a prediction framework, we propose that frontal activations found in said earlier studies (previously interpreted to refer to the FEF, attributed to oculomotor control and spatial attention) overlapped with prediction-related activation in adjacent parts of the PM. The current study aimed to provide preliminary evidence for this account.

In order to achieve this goal, our study had the following characteristics: (1) we developed a control condition (LUM) in a manner that allowed the application of identical visual input in both MOT and LUM conditions. The only difference between conditions was an initial task cue that did not enter the imaging analysis. (2) By asking participants to detect LUM as control condition, we intentionally designed a cognitive task that demanded to direct attention to the moving objects while allowing to disregard their trajectories. (3) Responses were required in both conditions, addressing the issue of activation in the DLFC due to response preparation (Jovicich et al. 2001). (4) We went to great lengths to identify and exclude FEF activation. Aside from stressing the importance to fixate on the fixation cross in order to reduce eye movements to a minimum, we conducted a prescreening that allowed us to select participants exhibiting the least visually guided saccades during MOT and LUM. Additionally, we functionally located participants’ FEF by recording brain activation during saccade execution (FEF-L). FEF-L was later applied as an exclusive mask to the MC.

### Results overview

#### Activations in the lateral frontal cortex

Corresponding to our hypothesis, the MC revealed bilateral frontal activation in BA6 comprising the precentral gyri and the precentral sulci, as well as left superior frontal gyrus (possibly merging into BA8), with the following cluster maxima (MNI, *x*/*y*/*z*): −15/−10/67 and 21/−10/61 (also see Table 1 and Fig. 2). As a rule of thumb, the threshold between the dorsal and the ventral PM lies in the range of *z*-coordinates 48–51 in Talairach space (Schubotz and von Cramon 2003; Tomassini et al. 2007), corresponding to approximately *z* = 43 to 46 in MNI space. Thus, we propose that this activation represents the involvement of premotor areas, namely the PMd. Noteworthy, further activations were found bilaterally in BA44 (pars opercularis of the IFG) with the following cluster maxima: 51/5/31 and −51/5/25 (also see Table 1 and Fig. 2). Even though these results did not reach the significance level of *P*_FDR-corrected_ < 0.001, these activations are of most interest to the current study, as we take them to reflect PMv involvement. Below, we will discuss these assumptions and speculate on the implications of our interpretations.

#### Activations in the temporal and parietal cortices

The MC revealed an extended activation cluster with local maxima in the superior and middle temporal gyri (bilateral), the right middle occipital gyrus, and the right supramarginal gyrus. This cluster spreads bilaterally through large parts of the parietal cortex (comprising the superior and inferior parietal lobules) and the occipital cortex (Table 1).

Similar parietal activations were found in previous studies (Culham et al. 1998, 2001; Jovicich et al. 2001; Howe et al. 2009). This area is generally associated with processes of spatial attention, for instance, governing attention shifts toward salient sensory input (Goodale and Milner 1992; Cabeza et al. 2008, 2011; Hutchinson et al. 2009; Sack 2009). The parietal cortex also comprises the parietal eye fields that are crucially involved in the execution of “reflexive” saccades toward salient objects in a visual scene (Rushworth et al. 2003; Pierrot-Deseilligny et al. 2004). Furthermore, the inferior parietal lobule, together with the IFG, has been associated with the embodiment of observed actions (Cross et al. 2009). On a similar note, parietofrontal circuits have been implicated to be involved in action planning and control by means of visuospatial and somatosensory representations (Wolpert and Ghahramani 2000; Lamm et al. 2007; Willems et al. 2009), even during *nonmotor* visuospatial mental operations, for example, mental rotations (Lamm et al. 2001).

Interestingly, previous brain imaging studies have not reported MOT-related activations in the temporal cortex that would resemble our findings. The superior temporal gyrus and sulcus have been associated with the attribution of animacy and mental states (Castelli et al. 2000). For instance, Schultz et al. (2004, 2005) used stimulus displays featuring abstract objects (geometrical shapes) that moved in an apparently self-propelled manner. The authors manipulated object “behavior” to give the impression of an “interaction” between two objects. They found activations in the superior and middle temporal gyrus in association with a high degree of attributed intentionality. We found activation maxima similar to those reported by Schultz and colleagues (our maxima: 54/−55/13, −57/−19/4, 42/−28/10; Schultz et al. 2004: 48/−44/12, −60/−56/4, −56/−30/4; Schultz et al. 2005: 39/−57/22; −60/−27/9). However, with the current experimental design, we cannot determine whether or not our participants may have attributed animacy and/or intentionality to the moving objects. Thus, the significance of our findings remains to be resolved by future studies.

In the following sections, we will focus our discussion on the activations in our area of interest, the frontal cortex.

### Dorsal and ventral premotor activations

In accordance with our hypothesis, we found activation maxima in BA6 and BA44. We assume that these activations reflect the involvement of the dorsal and ventral premotor cortices (PMd, PMv). The following sections will reflect on this assumption from anatomical and functional perspectives. Importantly, premotor activations would be in line with the idea of recruitment of prediction processes during MOT. However, alternative result interpretations will be addressed, namely processes of oculomotor control and visuospatial attention as the source of DLFC activation. We will conclude with speculations regarding the functional implications of our findings.

#### Functional boundaries of FEF versus PMd

Based on our finding of activation in the DLFC, the important question arises whether this activation can be attributed to the PMd, possibly representing prediction processes as hypothesized, or whether it should be rather attributed to FEF involvement governing oculomotor control. As the PMd and the FEF are adjacent (or even overlapping) brain structures (Melamed and Larsen 1979; Petit et al. 1996; Schubotz and von Cramon 2001; Ptak and Schnider 2011), this question cannot be easily answered based on anatomical parameters. To tackle this issue, we implemented the FEF-L, as described above. Following this procedure, we sought to functionally identify brain activations referring to eye movements. Another function that has been associated with FEF activation is processes of spatial attention (Corbetta 1998; Zacks et al. 2001). In an effort to exclude brain regions associated with these functions, we contrasted MOT against a control condition (LUM) that was designed as to engross similar cognitive resources (in regard to vigilance and attentional load) as MOT, as will be discussed below.

#### Oculomotor control and the DLFC

Oculomotor control during visual processing is often divided into two categories, referring to the origin of their initiation. Accordingly, eye movements can be labeled as *endogenous* (goal directed, cued, under top-down control, according to instruction) and *exogenous* (visually guided, noncued, under bottom-up control, stimulus driven). The involvement of the FEF in the execution of endogenous versus exogenous saccades has been subject to discussion (e.g., Anderson et al. 1994; Paus 1996; Pierrot-Deseilligny et al. 2004; Neggers et al. 2012).

By excluding FEF-L related activation from the MC, we sought to erase potential DLFC activation that might have been evoked by “accidentally executed” eye movements during MOT (i.e., despite the instruction to fixate on the fixation cross). Eye movements elicited by the FEF-L task were strongly exogenously driven (i.e., they were performed rapidly in response to target presentation). Accordingly, the application of the exclusive FEF-L mask to the MC removed possible brain activation associated with potential exogenous eye movements during MOT. Thus, any residual brain activation related to oculomotor control would point toward the occurrence of endogenous saccades during MOT. Indeed, while eye movements in the FEF-L task also bore *some* characteristics of endogenous saccades (i.e., there was a raised level of vigilance toward the appearance of targets in one of four possible locations), we cannot exclude the possibility that MOT elicited significantly more endogenous eye movements. Interestingly, one could argue, the execution of endogenous saccades toward a moving object would require a minimum degree of extrapolation of current object locations into the immediate future (and would thus support our prediction account). However, it is very unlikely (if at all) that accidental saccades in the MOT condition have occurred in a systematic manner such that they would have produced any contrast of relevance. In other words, they would have been prone to be eliminated as “noise” in the analyses. We are thus confident that neither exogenous nor endogenous saccades can account for the found DLFC activation.

Frontal eye fields activation has also been associated with continuous eye movements during smooth pursuit of target objects. Even so, we feel safe to exclude the occurrence of continuous eye movements, because Jovicich et al. (2001), who also conducted eye tracking during MOT, found no evidence of smooth pursuit, and neither did we during our behavioral prescreening.

However, the absence of saccade occurrences during MOT might point toward the employment of saccade *inhibition* processes (e.g., Culham et al. 1998). The significance of the FEF for the inhibition of exogenous, visually guided saccades has been a matter of debate. While there have been studies suggesting the FEF to be crucially involved in oculomotor-related inhibitory processes (Connolly et al. 2000; Kimmig et al. 2001; Pierrot-Deseilligny et al. 2004), it is noteworthy that the inhibition of exogenous saccades is usually measured by means of the “antisaccade paradigm.” This paradigm requires the performance of saccades toward the direction opposite to the locus of appearance of a visual object. Thus, result interpretation regarding the neural substrates of saccade inhibition based on this paradigm, where saccade suppression (toward the target), computation of the target's mirror position, and saccade execution (toward said mirror position) are confounded, is problematic. In line with this reasoning, there have been clinical findings painting a less clear picture of FEF involvement in inhibitory oculomotor control (Gaymard et al. 1999). A paradigm allowing for a more valid comparison with assumed eye movement inhibition in our MOT task would be saccade suppression during fixation with concurrently appearing peripheral visual stimuli. Neggers et al. (2012) tested this paradigm, the contrast of [Fixation with Peripheral Stimuli > Fixation without Peripheral Stimuli] revealing the following activation maxima (MNI, *x*/*y*/*z*): −38/−6/52, −52/0/38, 44/−2/52. As these activations are at the most tangentially overlapping with the activations found in the DLFC in our MC (maxima: −15/−10/67, 21/−10/61), we are confident that our allegedly found PMd activation did not originate from oculomotor suppression during visual fixation.

#### Spatial attention and the DLFC

Aside from oculomotor control, prior fMRI studies on MOT attributed activation in the DLFC to spatial attention during visual search (Culham et al. 1998, 2001; Jovicich et al. 2001; Howe et al. 2009). Indeed, brain activation related to spatial attention has been previously ascribed to the FEF (Corbetta 1998; Zacks et al. 2001), suggesting a strong link between the government of spatial attention and oculomotor control (“premotor theory of attention,” Rizzolatti et al. 1987). Other studies that found activation in the DLFC during the performance of spatial attention tasks have implicated the PMd as the region of origin. Boussaoud (2001), for instance, suggested that there are two subdivisions of the PMd, a rostral and a caudal part, that are rather distinct in regard to their functionality. While the caudal part appeared to be primarily involved in movement planning, the rostral part seemed to be mainly associated with the maintenance of spatial stimulus representations (Simon et al. 2002). Yet, arguably, the distinction between rostral PMd and posterior FEF cannot easily be made. In order to avoid a discussion of whether brain activation related to spatial attention originated in the PMd or the FEF, we are going to focus on functionality and use the term “areas in DLFC associated with spatial attention” (ADSA) in the following sections.

Aiming to address the issue of brain activation in the ADSA during MOT, we implemented a control condition (LUM). LUM required paying attention to the moving objects while disregarding their trajectories, as opposed to previous fMRI studies on MOT that used passive viewing control conditions (Culham et al. 1998, 2001; Jovicich et al. 2001; Howe et al. 2009). That is, in both conditions, participants had to attend to peripherally presented visual stimuli, and both conditions featured the same amount of objects that moved around in the same visual field (the *motion area*, roughly 7° of visual angle). As a consequence, we can assume that processes of spatial attention are considerably involved in both tasks. Thus, by contrasting MOT against LUM, we should have accounted for respective activation in the ADSA.

It is possible, though, that the two conditions differed in regard to spatial attentional *load*. While behavioral performance did not statistically differ, we cannot rule out this possibility. Rather, it appears to be intuitive to assume that MOT required more spatial attentional resources than LUM. However, Jovicich et al. (2001), who explicitly used the MOT paradigm in order to manipulate attentional load, did not find any load-related activations in the DLFC. That is, while possible differences in attentional load may have been manifest in other parts of the brain, we claim that it is unlikely that they can account for the activations in our target area.

A more specific component of spatial attention that might have elicited different amounts of ADSA activation in MOT compared to LUM is *shifts* in spatial attention. Just as eye movement control, attention shifts can be categorized as endogenous, goal directed and exogenous, sensory guided. The extent to which the ADSA are involved in both categories of spatial attention shifts is still under debate. For instance, Ptak and Schnider (2011) suggested that the ADSA are involved in both exogenous and endogenous attention shifts, whereas Corbetta and Shulman (2002) and Corbetta et al. (2005) claimed that the ADSA are rather responsible for endogenous, goal-directed attention shifts. In any case, remember that in the FEF-L task, upcoming target locations were visually guided (noncued), thus evoking exogenous shifts of attention. That is, after applying the exclusive FEF-L mask, any remaining attention-related activation in the MC can be ascribed to endogenous, goal-directed shifts in spatial attention. This interpretation would be in accordance with Yantis (1992), who proposed that maintenance of target identities is managed through top-down attention processes.

Interestingly, endogenous attention shifts and sensorimotor prediction processes are similar functional concepts (Bubic et al. 2010), insofar that they both act as internal generators bridging spatiotemporal information acquired in the immediate past (during exposure to the stimulus material) to current (and future) spatiotemporal stimulus characteristics. What remains to be resolved is the conceptual relation between the two. Is it possible that they are less separate processes as it might appear at first look? One approach to this question would be a critical review of cognitive tasks previously used to measure spatial attention shifts. What aspects of spatial attention were targeted with the respective tasks? To what extent might they have incorporated spatiotemporal extrapolation of target locations? Put differently, is it even possible to develop a cognitive paradigm able to disentangle processes of spatiotemporal prediction and spatial attention? Are the latter not rather a prerequisite for the former?

Unfortunately, these questions go way beyond the limits of the current study and will need to be addressed by future research. Importantly, if present, residual ADSA activation in the MC attributed to endogenous attention shifts would not contradict our idea that MOT involves cognitive mechanisms that provide internally guided (as opposed to externally triggered) processing of spatiotemporal information. However, the presence of such residual ADSA activation is highly speculative as we cannot determine if and how FEF-L, LUM, and MOT differed in respect to endogenous attention shifts.

Taken together, we propose that, after contrasting against LUM activation and subtracting FEF-L activation, we sufficiently accounted for regions in the DLFC that can be associated with components of oculomotor control and spatial attention similar to those occurring during MOT. Thus, we argue, the remaining activations in the MC represent those regions in the DLFC that are particularly involved in sensorimotor prediction, namely the PMd.

#### PMd activation

As outlined in the previous section, we suggest that the found activation maxima in the DLFC originated from PMd, possibly reflecting the involvement of prediction processes in MOT.

The engagement of the PM during tasks requiring the observation and imagination of others’ actions has gained considerable scientific attention (e.g., Grafton et al. 1997; Schubotz and von Cramon 2001; Decety and Grèzes 2006; Cross et al. 2009). In an fMRI study, the left PMd was interpreted to be “a core neural driver of action simulation” (Stadler et al. 2011, p. 677), for example, crucially contributing to the prediction of common routines (such as setting the dinner table) during 1000 msec occlusions (Stadler et al. 2011, 2012). However, the present study is by far not the first to associate this classic motor region with the prediction of inanimate dynamic visual events. For instance, the PMd has been associated with “spatially referenced” representations of action targets (Schubotz and von Cramon 2001, p. 98), as such serving the internal modeling of action sequences as well as perceptual events. Accordingly, PMd has been shown to be involved in a number of cognitive tasks requiring internal transformations of spatially defined perceptual events, such as serial prediction (Schubotz and von Cramon 2001), the generation of number sequences from memory (Abe et al. 2007), and mental rotation (Lamm et al. 2001; Oshio et al. 2010).

In this context, Schubotz (2007) suggested that (inanimate) event prediction is modulated by characteristic properties of the respective event, for example, “rhythmic” or “spatial.” Computations of corresponding forward models are processed in those premotor subareas whose regular motor output most suitably fits the respective event properties. Namely, Schubotz (2007; Schubotz and von Cramon 2003) proposed that prediction of spatially defined events is processed in dorsal premotor regions involved in reaching movements, while “object-defined events” are simulated by ventral premotor areas associated with grasping movements. Inanimate events are likely modulated by more than one salient property. Consequently, most inanimate events will evoke activations in more than one premotor subarea, as appears to be the case in the current study. Accordingly, we argue, the found PMd activation corresponds to the spatial emphasis of MOT.

#### PMv activation

The MC revealed bilateral activation in the pars opercularis of the IFG (BA44). This brain region has been most prominently associated with language production. However, recent research has also linked the pars opercularis to the processing of observed motor aspects (Rizzolatti and Craighero 2004). As a result, some authors have suggested that PMv extends from ventral BA6 into dorsal BA44 (Schubotz and von Cramon 2002, 2003; Schubotz et al. 2003; Binkofski and Buccino 2006).

BA44 has been argued to be the putative human homologue of the monkey premotor area F5 (Petrides et al. 2005) in which so-called “mirror neurons” were observed (Gallese et al. 1996). It appears that neurons in this area code sensorimotor representations, presumably in a modality-independent way (Bremmer et al. 2001). Interestingly, in monkeys, these neurons also fire when action goals (e.g., object contact as the goal of a reach-to-grasp movement) are occluded (Umiltà et al. 2001), indicating their predictive capacities. Similarly, it has been suggested that, together with BA6 and parietal areas (BA2), human BA44 is part of right hemisphere pathways that, aside from multisensory processing, are assumed to provide forward models based on somatosensory representations and sensorimotor consequences of planned or simulated actions (Wolpert and Ghahramani 2000; Lamm et al. 2007; Willems et al. 2009).

Furthermore, the human opercular part is often associated with complex and abstract action-related cognition. Such forms of action representations are, for instance, required for multilimb coordination in complex movements (Swinnen et al. 2010), as well as during the observation of such movements (Calvo-Merino et al. 2006; Cross et al. 2006). Abstract action representations involve the encoding of complex rules for spatiotemporal organization among movements of single limbs. Moreover, pars opercularis in the left hemisphere has been demonstrated to be engaged in chunking, enabling the construction of hierarchical structures in language and mathematics (Makuuchi et al. 2012). Thus, functions of the opercular part are recruited not only during action production and observation, but also in cognitive tasks that require the establishment of complex rules for spatiotemporal organization. Accordingly, it can be speculated that the bilateral activation in pars opercularis found in the MC reflects the occurrence of rule detection, enabling mental representations of spatial relations between the tracked objects. Such mental representations may, for instance, involve the structuring of spatial information into chunks. Indeed, Yantis (1992) found empirical evidence suggesting that participants in an MOT task showed forthwith mental grouping of targets as if they belonged to one bigger object (also see above, Cognitive processes during MOT). When maintenance of such a cognitive representation was experimentally disrupted, tracking performance was impaired.

Activation in the inferior frontal cortex has been previously associated with MOT (Culham et al. 1998), more precisely with parametric tracking effects (Culham et al. 2001; Jovicich et al. 2001). In order to test for MOT-specific load components, we conducted an explorative analysis, comparing brain activation during the tracking of three compared to two objects, [MOT3 > MOT2] (*P*_uncorrected_ < 0.05; voxel threshold *k* = 10). In an attempt to control for activation related to general attentional load, we applied activation of the contrast [LUM2 > LUM1] (*P*_uncorrected_ < 0.05; voxel threshold *k* = 10) as an exclusive mask. This procedure revealed activation in the pars opercularis of the right IFG (cluster maximum in MNI, *x*/*y*/*z*: 51/8/28). The lack of a more pronounced MOT-specific load activation can be attributed to the fact that we had only two levels of difficulty. Jovicich et al. (2001), for instance, found a linear increase of activation in the inferior precentral sulcus (possibly referring to BA44) with increasing number of two to five tracked objects. In contrast, our manipulation of task difficulty might not have been powerful enough to yield more significant brain activations associated with MOT-specific processing load.

However, note that these speculations are based on the assumption that we did manage to account for activations due to attentional load by contrasting against LUM. While we did not find statistical differences in behavioral performance in MOT compared to LUM, it has to be acknowledged that the amount of correct answers might not be an ideal measure for attentional load. Rather, MOT might have strained attention processes to a different extent than LUM. Thus, attentional load elicited by MOT, as suggested by Jovicich et al. (2001), remains a reasonable explanation for the found activations in the pars opercularis. Future studies will have to address this issue.

#### Implications of PM activation

“Predictions that allow one to anticipate features such as the movements of objects and the behaviors of other animals are of great adaptive benefit” (Zacks et al. 2011, p. 4057). More precisely, predictions of dynamic perceptual events are a prerequisite for goal-directed manipulations of and beneficial reactions to social and physical environments. For instance, only through the prediction of biological movements are we able to successfully engage in cooperative or competitive interactions with conspecifics.

Against this background, let us consider a tangible example to understand the real world significance of the abstract MOT paradigm. Picture a herd of animals. A predator observing the herd is keen to single out and keep track of its weakest member. From the observer's perspective, this individual animal, as it trots about, is repeatedly occluded by trees, rocks, or other animals. Its bodily outline is in constant change while it adjusts its movement directions. Changes in lighting can lead to variations in optical refractions, resulting in the animal's fur to be perceived in different colors. Transferring this scene to the MOT paradigm, behavioral results suggest that the human brain is well adapted to compensate for such fluctuations in visual input during tracking (Scholl and Pylyshyn 1999; Bahrami 2003). Importantly, in the presence of nondiscriminatory or ambiguous object surface features, the continuity of target identities appears to strongly rely on spatiotemporal information, such as motion trajectories (Franconeri et al. 2006). We propose that motion trajectories are not only processed up to the point of current target locations, but that their future courses are extrapolated via sensorimotor anticipation processes (Chaminade et al. 2001).

Previous brain imaging studies provided evidence of the PM to be a neural correlate of the prediction of familiar human actions (Stadler et al. 2011, 2012) and inanimate events (Schubotz 2007; Wolfensteller et al. 2007). Accordingly, PM activations in the current study are in line with the idea that sensorimotor prediction processes were also recruited during MOT. This finding could indicate that, during the parallel tracking of inanimate entities performing arbitrary motions, prediction processes are employed similar to those used to pursue and anticipate goal-directed movements of biological agents. Although this interpretation is pure speculation at this point, this would not be the first study to report PM activation during the prediction of unfamiliar, arbitrary movement. Cross et al. (2011a), for instance, found PM involvement when participants were asked to predict continuations of action sequences that did not match their own motor expertise (gymnastic sequences). This was also true for predictions of inanimate toy movements (wind-up toys, Cross et al. 2011a).

At first glance, the employment of prediction processes during (rather “unpredictable”) nonbiological, arbitrary perceptual events might appear maladaptive, as they are bound to lead to guesswork. Yet, only through such initial guesswork can a feedback process be launched (Van der Stigchel et al. 2009) that has the potential to eventually lead to the acquisition of new (predictive) sensorimotor experience (cf. Cross et al. 2006). Thus, we suggest that the human brain's tendency to employ prediction processes, even during the observation of unfamiliar, arbitrary, or nongoal-directed movements (cf. Cross et al. 2006, 2011a,b), is of vital adaptive advantage (cf. Bubic et al. 2010).

### Summary

The current study aimed to investigate the recruitment of prediction processes during the tracking of abstract objects following arbitrary motion trajectories (MOT; Pylyshyn and Storm 1988). We operated under the assumption that prediction processes should be reflected by PM activation, as the PM has been previously demonstrated to be significantly involved in predictions of perceptual and motor events (Schubotz and von Cramon 2004; Schubotz 2007; Wolfensteller et al. 2007; Stadler et al. 2011, 2012).

Recording fMR-images during the performance of an MOT task, we replicated previous results (Culham et al. 1998, 2001; Jovicich et al. 2001; Howe et al. 2009), revealing activations in occipitotemporal, parietal, and frontal areas. We claim that the found activations in the frontal cortex represent the dorsal and ventral premotor cortices. Importantly, though the role of cognitive resources other than prediction processes cannot be exhaustively determined, we made an effort to develop an experimental design that – to a considerable extent – was able to account for frontal activations associated with oculomotor control and spatial attention processes.

To conclude, we propose that the found activations in the PM point toward a signature of sensorimotor predictions of motion trajectories during MOT.
